# Supplementary material for: Enhanced specificity of Bacillus metataxonomics using a tuf-targeted amplicon sequencing approach
Source: ISME Commun. 2023 Nov 27;3:126. doi: 10.1038/s43705-023-00330-9 (PMC10682494; doi:10.1038/s43705-023-00330-9)
Supplement: Supplementary file 1 — Supplementary information [file 43705_2023_330_MOESM1_ESM.pdf]

Dataset S1 List of *Bacillus* genomes downloaded from NCBI in April 2022

Dataset S2 Suggested primers for *tuf*, *gyrA*, and *rpoB* loci

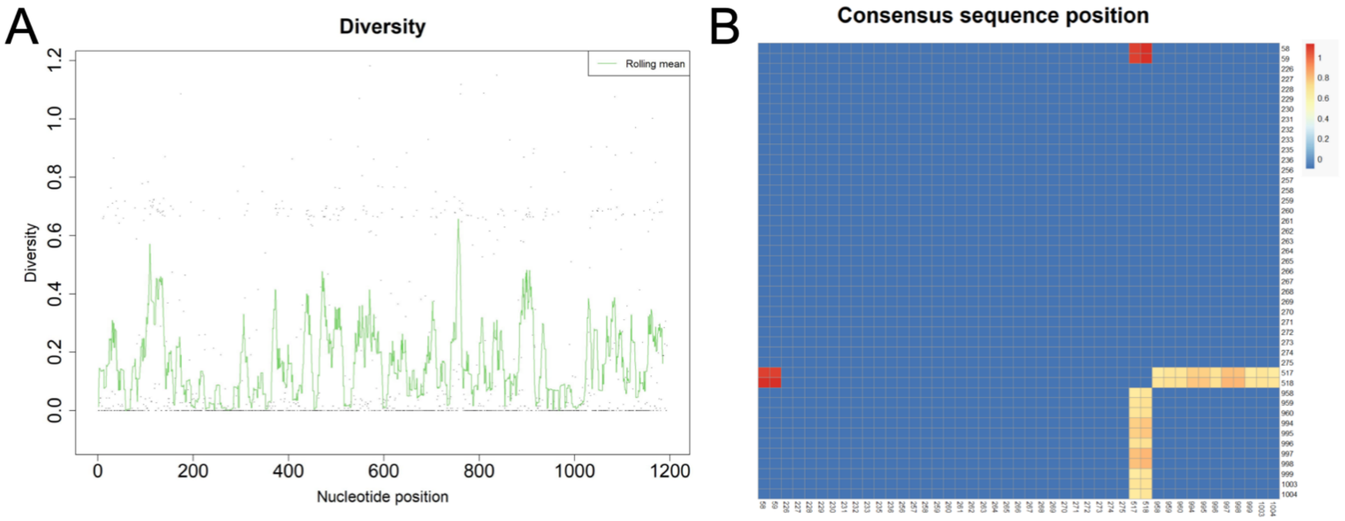

Fig. S1 Selecting primer positions in the *tuf* gene. **A** Nucleotide entropy profile in a alignment of tuf genes derived from *Bacillus* genome collection. **B** Highly scoring primer position corresponding to the entropy profile of the *tuf* gene alignment.

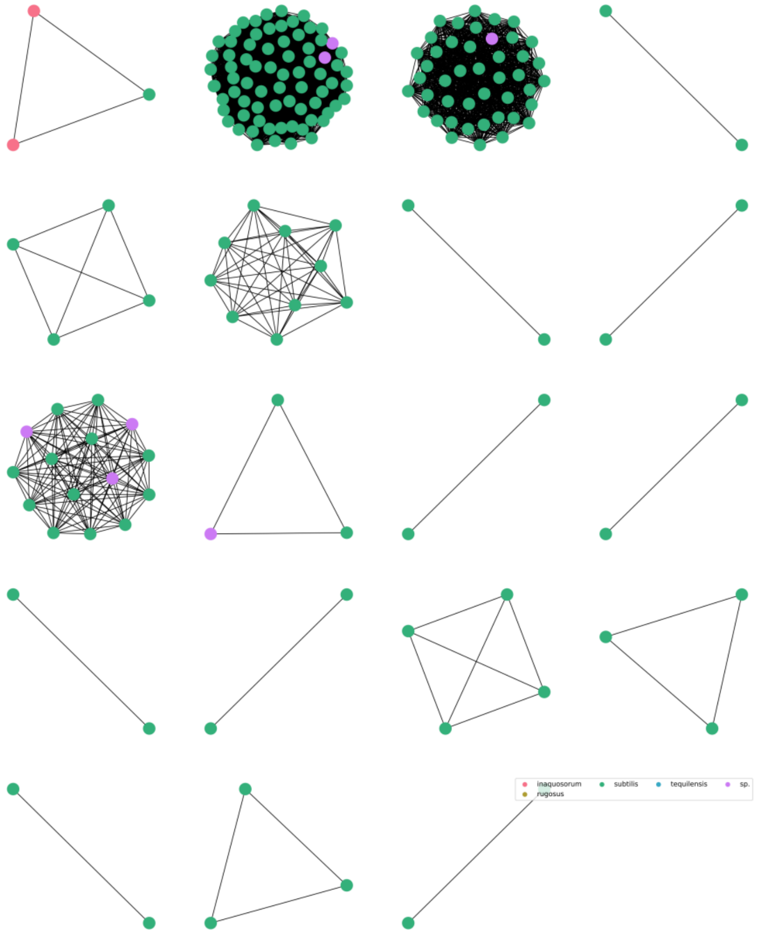

Fig. S2 Network visualization of amplicons derived from *gyrA-42f* - *gyrA-1066r* primer sets. Nodes represented *Bacillus* genomes and were connected if amplicon derived from these genomes have overlapping alleles. Node color described different *Bacillus* species. Non-connected nodes are excluded. Edge width is proportion to the number of shared alleles.

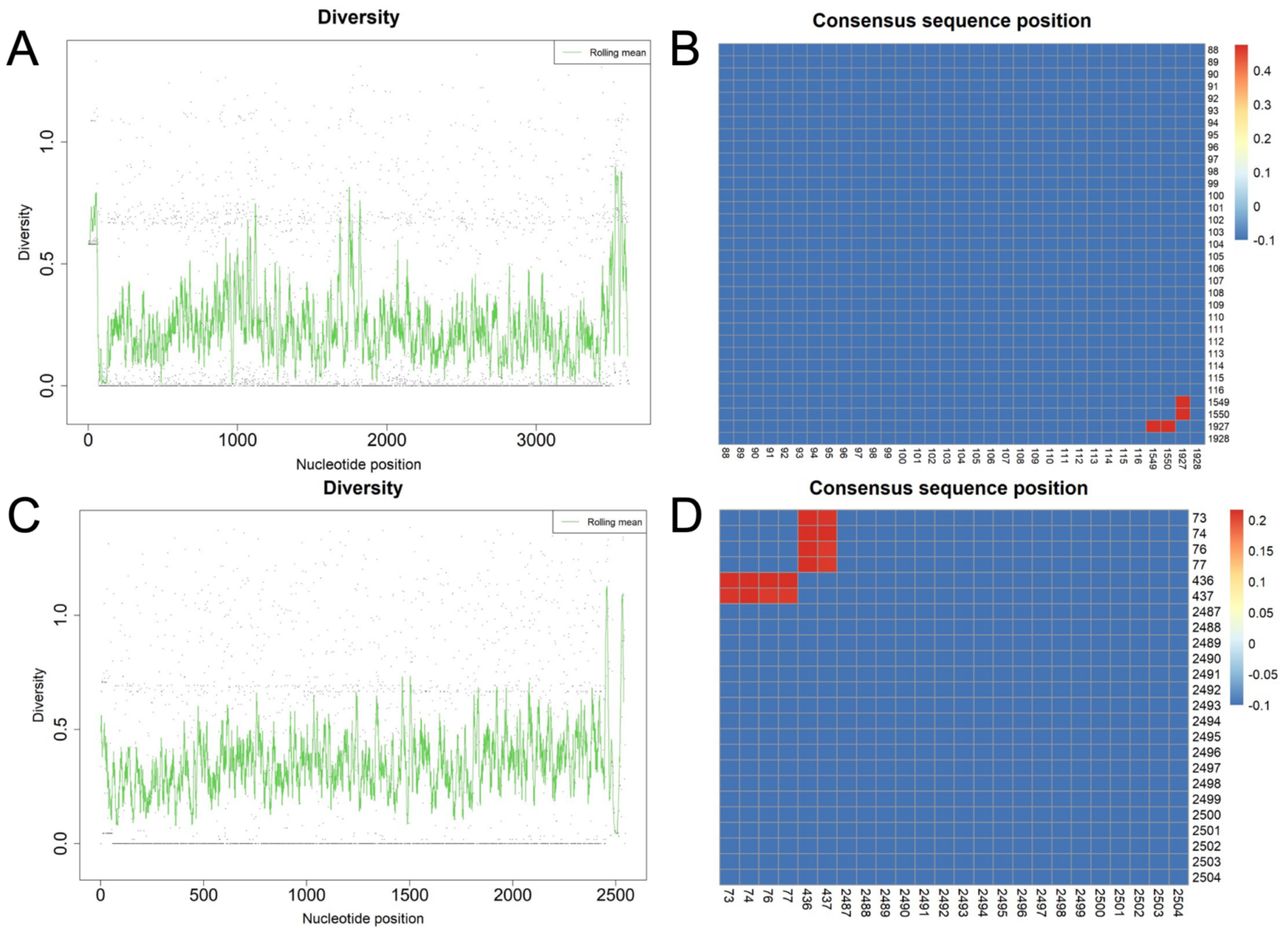

Fig. S3 Selecting primer positions in the *rpoB* and *gyrA* gene.**A** Nucleotide entropy profile in *rpoB* gene alignment. **B** Highly scoring primer position corresponding to the entropy profile of the *rpoB* gene alignment. **C** Nucleotide entropy profile in *gyrA* gene alignment. **D** Highly scoring primer position corresponding to the entropy profile of the *gyrA* gene alignment.

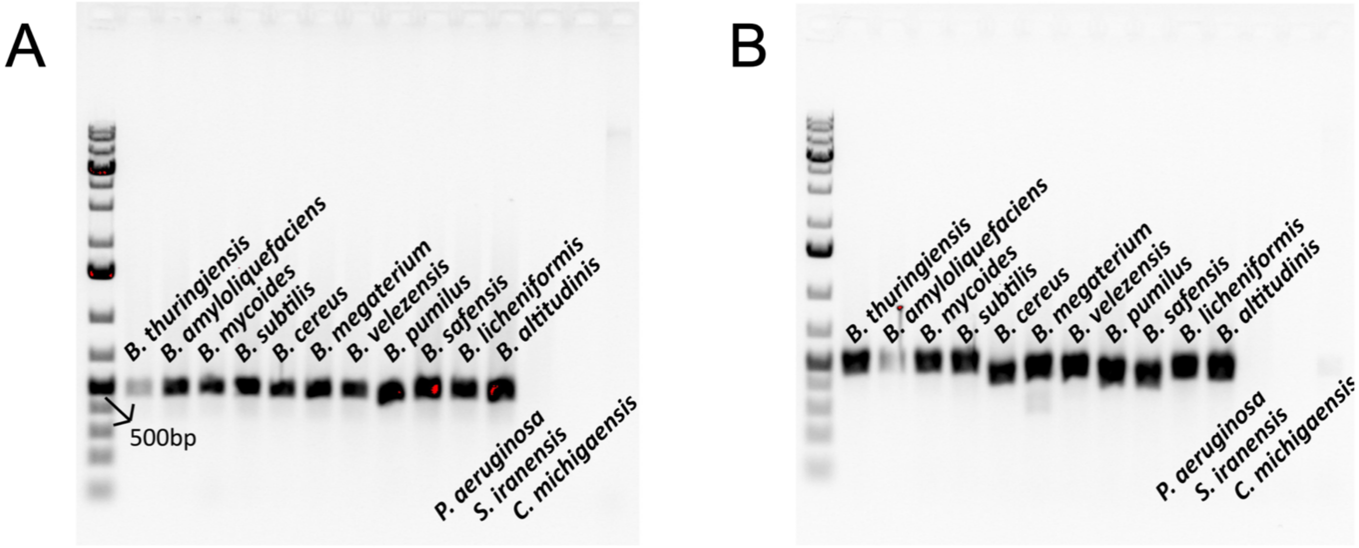

Fig. S4 Agarose gel electrophoresis of *tuf* gene PCR products amplified from members of Bac-DNAmix using *tuf1* and *tuf2* primers. The last three lanes are non-*Bacillus* strains *Pseudomonas aeruginosa, Streptomyces iranensis, and Clavibacter michiganensis* used as negative controls. Image has been contrast enhanced for better visibility of any band produced. The size marker is Gene ruler 1 kb DNA Ladder (Thermo Fisher Scientific Catalog #SM0311).

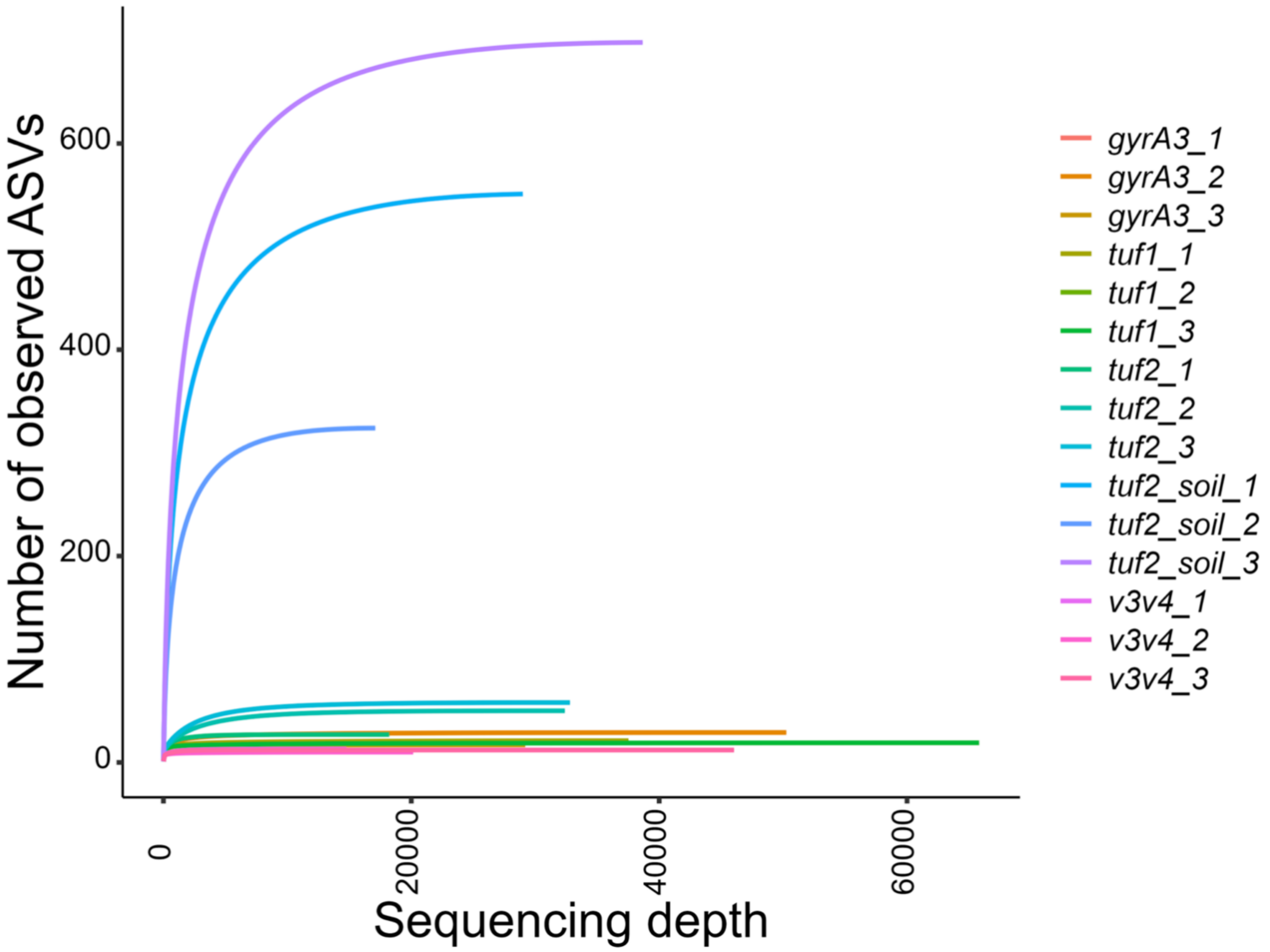

Fig. S5 Rarefaction curve for each sample and replicates.

Table S1 List of barcodes tagged on primers for Illumina sequencing of amplicons of the *Bacillus tuf* gene.

**Sample**	**Barcode**
tuf1_1	TTTTAATC
tuf1_2	ATAATTAG
tuf1_3	ACCAAATT
tuf2_1	CTTATCAA
tuf2_2	TGATCATT
tuf2_3	AGAATCTA
gyrA3_1	TCAAGAAA
gyrA3_2	ATCGAAAT
gyrA3_13	ACATTTAC
tuf2_soil_1	TAGAAAAC
tuf2_soil_2	TTATCACC
tuf2_soil_3	AATAGGGT
v3v4_1	GAGAGGGA
v3v4_2	ATCCCGGT
v3v4_3	GAGCTCCT

Table S2: List of non-*Bacillus* genomes tested in silico to validate the specificity of *Bacillus* amplicon primers.

Phyla/Division	Species	Strain	RefSeq accession
Actinobacteria	*Bifidobacterium bifidum*	S6	GCF_003390735.1
Actinobacteria	*Clavibacter michiganensis*	UF1	GCF_009739655.1
Actinobacteria	*Mycobacterium tuberculosis*	HN-506	GCF_002357975.1
Actinobacteria	*Micrococcus luteus*	CW.Ay	GCF_019890915.1
Actinobacteria	*Rhodococcus globerulus*	D757	GCF_019334125.1
Actinobacteria	*Streptomyces coelicolor*	A3(2)	GCF_000203835.1
Actinobacteria	*Streptomyces iranensis*	DSM 41954	GCF_017874715.1
Alphaproteobacteria	*Azospirillum brasilense*	Az39	GCF_000632475.1
Alphaproteobacteria	*Agrobacterium tumefaciens*	Ach5	GCF_000971565.1
Alphaproteobacteria	*Bradyrhizobium diazoefficiens*	110spc4	GCF_004359355.1
Ascomycota	*Alternaria alternate*	SRC1lrK2f	GCF_001642055.1
Ascomycota	*Fusarium oxysporum*	NRRL 32931	GCF_000271745.1
Ascomycota	*Saccharomyces cerevisiae*	S288C	GCF_000146045.2
Betaproteobacteria	*Achromobacter xylosoxidans*	AX1	GCF_008432465.1
Betaproteobacteria	*Achromobacter xylosoxidans*	MN001	GCF_001051055.1
Betaproteobacteria	*Bordetella pertussis*	H640	GCF_004008975.1
Betaproteobacteria	*Neisseria meningitidis*	7-Nov	GCF_008330805.1
Deltaproteobacteria	*Myxococcus xanthus*	DK 1622	GCF_000012685.1
Firmicutes	*Aneurinibacillus migulanus*	DSM 2895	GCF_001274715.1
Firmicutes	*Clostridium acetobutylicum*	ATCC 824	GCF_000008765.1
Firmicutes	*Lactococcus lactis*	LAC460	GCF_020463755.1
Firmicutes	*Paenibacillus amylolyticus*	FSL H7-0692	GCF_001956035.1
Firmicutes	*Paenibacillus graminis*	DSM 15220	GCF_000758705.1
Firmicutes	*Paenibacillus polymyxa*	ZF129	GCF_006274405.1
Firmicutes	*Staphylococcus aureus*	NCTC 8325	GCF_000013425.1
Firmicutes	*Staphylococcus epidermidis*	NIHLM061	GCF_000276445.1
Flavobacteriia	*Chryseobacterium sp.*	D764	GCF_019317325.1
Gammaproteobacteria	*Acinetobacter baumannii*	K09-14	GCF_008632635.1
Gammaproteobacteria	*Enterobacter hormaechei subsp. xiangfangensis*	ND10	GCF_000784035.1
Gammaproteobacteria	*Legionella pneumophila*	C9_S	GCF_001753085.1
Gammaproteobacteria	*Pseudomonas aeruginosa*	PAO1	GCF_000006765.1
Gammaproteobacteria	*Pseudomonas fluorescens*	ATCC 13525	GCF_900215245.1
Gammaproteobacteria	*Pseudomonas koreensis*	LMG21318	GCF_900101415.1
Gammaproteobacteria	*Pseudomonas moraviensis*	TYU6	GCF_002287825.1
Gammaproteobacteria	*Pseudomonas protegens*	CHA0	GCF_900560965.1
Gammaproteobacteria	*Pseudomonas stutzeri*	F2a	GCF_019704535.1
Gammaproteobacteria	*Stenotrophomonas indicatrix*	D763	GCF_019285675.1
Gammaproteobacteria	*Vibrio parahaemolyticus*	O3:K6 substr. RIMD 2210633	GCF_000196095.1
Nematoda	*Caenorhabditis elegans*	Bristol N2	GCF_000002985.6
Sphingobacteria	*Sphingobacterium sp.*	B29	GCF_001952815.1
Sphingobacteria	*Pedobacter sp.*	D749	GCF_019317285.1

Table S3: Annotation results of 20 soil-derived isolates derived from NCBI using 16S rRNA and *tuf2* primer pairs. Species names were assigned based on the complete genomes.

Species name	Strain	16S blast results	Per. Ident	*tuf2* blast results	Per. Ident
Bacillus pumilus	B2_10	*Peribacillus butanolivorans*	99.79%	*Bacillus pumilus*	98.25%
Bacillus subtilis	C1_13	*Bacillus tequilensis*	99.51%	*Bacillus subtilis*	95.37%
Bacillus subtilis	C1_9	*Bacillus sp.*	98.58%	*Bacillus subtilis*	99.14%
Bacillus velezensis	Canada_S1	*Bacillus sp. (in: Bacteria)*	99.65%	*Bacillus velezensis*	99.34%
Peribacillus simplex	D8_B_37	*Lysinibacillus sp.*	98.12%	*Peribacillus simplex*	98.91%
Bacillus licheniformis	D9_B_45	*Bacillus licheniformis*	99.93%	*Bacillus licheniformis*	99.12%
Bacillus altitudinis	D9_B_49	*Bacillus sp. (in: Bacteria)*	99.93%	*Bacillus altitudinis*	99.34%
Bacillus subtilis	D9_B_56	*Bacillus sp. (in: Bacteria)*	99.86%	*Bacillus subtilis*	98.92%
Peribacillus simplex	D9_B_73	*Peribacillus simplex*	99.65%	*Brevibacterium sp. PAMC21349*	98.46%
Peribacillus simplex	E1_1	*Peribacillus simplex*	100.00%	*Brevibacterium sp. PAMC21349*	98.46%
Peribacillus frigoritolerans	G1S1	*Bacillus sp. (in: Bacteria)*	99.93%	*Brevibacterium sp. PAMC21349*	98.91%
Bacillus subtilis	G2S2	*Bacillus subtilis*	97.79%	*Bacillus subtilis*	99.34%
Bacillus altitudinis	G6S2	*Bacillus pumilus*	99.72%	*Bacillus altitudinis*	98.48%
Bacillus safensis	G6S3	*Bacillus sp. (in: Bacteria)*	99.86%	*Bacillus safensis*	98.91%
Bacillus pumilus	Monterrea_S2	*Bacillus pumilus*	99.86%	*Bacillus pumilus*	98.65%
Bacillus velezensis	MB7_B13	*Bacillus velezensis*	99.65%	*Bacillus velezensis*	98.11%
Bacillus halotolerans	KF17	*Bacillus sp. C26*	99.72%	*Bacillus halotolerans*	99.12%
Priestia megaterium	AQ13	*Rossellomorea aquimaris*	99.93%	*Priestia megaterium*	98.71%
